# Cancer risk and tumour spectrum in 172 patients with a germline *SUFU* pathogenic variation: a collaborative study of the SIOPE Host Genome Working Group

**DOI:** 10.1136/jmedgenet-2021-108385

**Published:** 2022-06-29

**Authors:** Léa Guerrini-Rousseau, Julien Masliah-Planchon, Sebastian M Waszak, Pia Alhopuro, Patrick R Benusiglio, Franck Bourdeaut, Ines B Brecht, Giada Del Baldo, Sandeep Kumar Dhanda, Maria Luisa Garrè, Corrie E M Gidding, Steffen Hirsch, Pauline Hoarau, Mette Jorgensen, Christian Kratz, Lucie Lafay-Cousin, Angela Mastronuzzi, Lorenza Pastorino, Stefan M Pfister, Christopher Schroeder, Miriam Jane Smith, Pia Vahteristo, Roseline Vibert, Catheline Vilain, Nicolas Waespe, Ingrid M Winship, D Gareth Evans, Laurence Brugieres

**Affiliations:** 1 Department of Children and Adolescents Oncology, Gustave Roussy, Villejuif, France; 2 Team “Genomics and Oncogenesis of pediatric Brain Tumors”—Paris Saclay University, INSERM U981, VILLEJUIF, France; 3 INSERM U830, Laboratory of Translational Research in Pediatric Oncology, SIREDO Pediatric Oncology Center, Institute Curie, Paris, France; 4 Centre for Molecular Medicine Norway (NCMM), Nordic EMBL Partnership, University of Oslo and Oslo University Hospital, Oslo, Norway; 5 Department of Pediatric Research, Oslo University Hospital, Oslo, Norway; 6 Department of Medical and Clinical Genetics, University of Helsinki, Helsinki, Finland; 7 Département de Génétique et Institut Universitaire de Cancérologie, Sorbonne University Faculty of Medicine Pitié-Salpêtrière Campus, Paris, France; 8 Department of Pediatric Oncology and Hematology, University Hospitals Tubingen, Tubingen, Germany; 9 Department of Hematology/Oncology, Cell Therapy, Gene Therapy and Hemopoietic Transplant, IRCCS, Bambino Gesu Pediatric Hospital, Roma, Italy; 10 Department of Oncology, St Jude Children's Research Hospital, Memphis, Tennessee, USA; 11 Neuro-Oncology Unit, Department of Neurochirurgia, IRCCS Istituto Giannina Gaslini, Genova, Italy; 12 Neuro-Oncology Department, Princess Maxima Center for Pediatric Oncology, Utrecht, The Netherlands; 13 Institute of Human Genetics, University Hospital Heidelberg, Heidelberg, Germany; 14 Hopp Children's Cancer Center Heidelberg (KiTZ), Heidelberg Health Center, Heidelberg, Germany; 15 Oncology, Great Ormond Street Hospital For Children NHS Foundation Trust, London, UK; 16 Paediatric Haematology and Oncology, Hannover Medical School, Hannover, Germany; 17 Section of Pediatric Hematology Oncology and Bone Marrow Transplantation, Alberta Children's Hospital and Cumming School of Medicine, University of Calgary, Calgary, Alberta, Canada; 18 Pediatric Hematology/Oncology and Stem Cells Transplatation, Bambino Gesu Pediatric Hospital, Roma, Italy; 19 Department of Oncology, Biology and Genetics, University of Genoa, Genoa, Italy; 20 Genetics of Rare Cancers, IRCCS Ospedale Policlinico San Martino, Genova, Italy; 21 Division of Pediatric Neurooncology, DKFZ, Heidelberg, Germany; 22 Department of Pediatric Oncology, Hematology and Immunology, University Hospital Heidelberg, Heidelberg, Germany; 23 Institute of Medical Genetics and Applied Genomics, University of Tubingen Institute of Human Genetics, Tubingen, Germany; 24 Division of Evolution, Infection and Genomics, The University of Manchester, Manchester, UK; 25 Department of Medical and Clinical Genetics, Applied Tumor Genomics Research Program, University of Helsinki, Helsinki, Finland; 26 Department of Genetics, PSL Research University, Institute Curie, Paris, France; 27 Department of Genetics, Hôpital Universitaire des Enfants Reine Fabiola, ULB Center of Human Genetics, Universite Libre de Bruxelles, Bruxelles, Belgium; 28 Department of Genetics, Hôpital Erasme, ULB Center of Human Genetics, Universite Libre de Bruxelles, Bruxelles, Belgium; 29 CANSEARCH Research Platform, Depatment of pediatric oncology and hematology, University of Geneva, Geneva, Switzerland; 30 Childhood Cancer Research Group, Institute of Social and Preventive Medicine, University of Bern, Bern, Switzerland; 31 Department of Medicine, The Royal Melbourne Hospital, Parkville, Victoria, Australia; 32 Manchester Centre for Genomic Medicine, St Mary's Hospital, Manchester Academic Health Science Centre, School of Biological Sciences, Division of Evolution, Infection and Genomics, The University of Manchester, Manchester, UK; 33 Department of Children and Adolescents Oncology, Gustave Roussy Institute, Villejuif, France

**Keywords:** genetic predisposition to disease, germ-line mutation, central nervous system diseases, genetic counseling, congenital, hereditary, and neonatal diseases and abnormalities

## Abstract

**Background:**

Little is known about risks associated with germline *SUFU* pathogenic variants (PVs) known as a cancer predisposition syndrome.

**Methods:**

To study tumour risks, we have analysed data of a large cohort of 45 unpublished patients with a germline *SUFU* PV completed with 127 previously published patients. To reduce the ascertainment bias due to index patient selection, the risk of tumours was evaluated in relatives with *SUFU* PV (89 patients) using the Nelson-Aalen estimator.

**Results:**

Overall, 117/172 (68%) *SUFU* PV carriers developed at least one tumour: medulloblastoma (MB) (86 patients), basal cell carcinoma (BCC) (25 patients), meningioma (20 patients) and gonadal tumours (11 patients). Thirty-three of them (28%) had multiple tumours. Median age at diagnosis of MB, gonadal tumour, first BCC and first meningioma were 1.5, 14, 40 and 44 years, respectively. Follow-up data were available for 160 patients (137 remained alive and 23 died). The cumulative incidence of tumours in relatives was 14.4% (95% CI 6.8 to 21.4), 18.2% (95% CI 9.7 to 25.9) and 44.1% (95% CI 29.7 to 55.5) at the age of 5, 20 and 50 years, respectively. The cumulative risk of an MB, gonadal tumour, BCC and meningioma at age 50 years was: 13.3% (95% CI 6 to 20.1), 4.6% (95% CI 0 to 9.7), 28.5% (95% CI 13.4 to 40.9) and 5.2% (95% CI 0 to 12), respectively. Sixty-four different PVs were reported across the entire *SUFU* gene and inherited in 73% of cases in which inheritance could be evaluated.

**Conclusion:**

Germline *SUFU* PV carriers have a life-long increased risk of tumours with a spectrum dominated by MB before the age of 5, gonadal tumours during adolescence and BCC and meningioma in adulthood, justifying fine-tuned surveillance programmes.

**What is already known on this topic**

- Germline *SUFU* pathogenic variant (PV) was described for the first time associated with the occurrence of medulloblastoma by Michael Taylor *et al* in 2002.
- Before our study, germline SUFU PVs were known to be associated with a cancer predisposition syndrome predisposing to SHH-medulloblastoma during the first 3 years of life as well as cancers associated with Gorlin syndrome.
- During the last years, >100 patients with a germline SUFU PV have been reported, but most often, these publications are case reports in which the SUFU PV was identified after the occurrence of cancer.
- Due to the rarity of this clinical situation, little was known about tumour risks and outcome of patients in this condition.

**What this study adds**

- Germline SUFU PV carriers have a life-long increased risk of tumours.
- Data from this large series allow describing the oncological spectrum of SUFU PVs, dominated by medulloblastoma before the age of 5 years, gonadal tumors during adolescence and basal cell carcinoma and meningioma in adulthood.
- We also aimed to evaluate cancer risk, but as most index patients have been identified after the occurrence of a malignancy, we analysed the cumulative risk of tumour in relatives only, after exclusion of the index cases in order to reduce bias.
- We were able to confirm that the tumour penetrance (any type of tumour) is high, although incomplete reaching 44% at 50 years.

**How this study might affect research, practice and/or policy**

- Thanks to this large international cooperation, we could describe the spectrum of tumours, the cumulative risk of cancer as well as the period of onset of each tumour type during life associated with a germline SUFU PV.
- These information allow designing guidelines for PV carriers follow-up based on comprehensive data.

## INTRODUCTION

Gorlin syndrome (GS) (MIM 109400), or nevoid basal cell carcinoma syndrome (NBCCS), is an autosomal dominantly inherited syndrome characterised by developmental anomalies including macrocephaly, frontal bossing, hypertelorism and has been described as a cancer predisposition syndrome.^1 2^ The tumour spectrum includes malignant tumours, mostly basal cell carcinomas (BCC) and medulloblastomas (MB), and benign tumours such as keratocystic odontogenic tumours, meningiomas, ovarian or cardiac fibromas.^1 3–5^ Most individuals affected by GS have a heterozygous germline pathogenic variant (PV) in Sonic Hedgehog pathway genes: Patched 1 (*PTCH1*)^6 7^ or Suppressor of fused (*SUFU*).^8 9^ A GS-like clinical presentation has been recently described in children with heterozygous germline *GPR161* variants.^10^ The role of Patched 2 (*PTCH2*)^11 12^ in the pathogenesis of GS has also been suggested and questioned.^13^

Defining the incidence and spectrum of tumours in *SUFU-*associated GS is complicated due to the paucity of information collected so far. The association of germline *SUFU* PVs and nodular desmoplastic MB was described for the first time in 2002 by Taylor *et al*.^14^ Since then, most information we have on germline *SUFU* mutation carriers comes from patients identified after the occurrence of a tumour, mainly MB. A few *SUFU* mutation carriers have also been identified after the occurrence of a meningioma^15 16^ and cutaneous cancers.^17 18^ Additional information comes from cohorts of patients presenting the clinical characteristics of GS in whom 5% are identified with a *SUFU* PV.^19^ In a large series of 1022 patients with MB analysed for germline variants,^20^ 6% of the patients were found to carry a germline PV in a known cancer predisposition gene, including 11 patients (1.1%) with a germline *SUFU* PV and 9 patients (0.9%) with a germline *PTCH1* PV, all with SHH-activated MB (SHH-MB).^21 22^ The prevalence of germline *SUFU* or *PTCH1* PVs in SHH-MB below the age of 3 years was 21%.^20^

In recent years, >100 patients with a germline *SUFU* PV have been reported,^8 9 14 19 20 23–36^ mainly as case reports; but data quantifying tumour risk and outcome of these patients remain scarce. The recent creation of the Host Genome Working Group (HGWG) in the European branch of the International Society of Pediatric Oncology (SIOPE) aimed at improving care for patients with paediatric cancer predisposition syndromes. The SIOPE-HGWG allowed us to set up a large international collaboration to increase the knowledge on this predisposition syndrome. The objective of this study was to describe the tumour spectrum and cancer risks specifically associated with germline *SUFU* PVs in order to provide recommendations to affected patients and their family members based on a comprehensive cohort.

## Patients and methods

### Inclusion criteria

This study includes only individuals with a germline PV in the *SUFU* gene referred to as *SUFU* PV carriers. We analysed the literature to collect all patients with a *SUFU* PV in articles published before 1 January 2021. We contacted the authors of these publications to obtain follow-up information. In addition, through the SIOPE-HGWG, we also collected data of unpublished patients from eight different countries.

### Data collection

For all *SUFU* PV carriers, we collected data on tumours identified so far and vital status at the last follow-up. In each family, the first patient in whom the PV was identified was defined as the index patient, whether she/he had a tumour or not. All their family members, in whom the *SUFU* PV was identified after its identification in the index patient, were qualified as relatives. Data from French patients, currently collected in the French ‘Observatory of Genetic Cancer Predisposition Syndromes in Children and Adolescents’ (Observatoire des syndromes de prédisposition génétique au cancer des enfants et des adolescents, PREDCAP, IRB00003888) were merged with data obtained from each national group.

### Statistics

Baseline values (ie, at diagnosis) were expressed as medians and ranges for continuous variables, and as numbers and percentages for categorical variables, and compared using the χ^2^ test. Overall survival (OS) rates were calculated using the Kaplan-Meier method. Overall survival time after MB was estimated from the date of diagnosis of the MB to death, whatever the cause, or the date of the last follow-up. The 95% CI values for OS rates were estimated with the Rothman method. The Nelson and Aalen estimator^37^ was used to model the cumulative incidence curves in relatives carrying the *SUFU* PV. To study genotype-phenotype correlation, the impact of the type of the *SUFU* PV on the risk of MB was estimated using the χ^2^ test, and p values <0.05 were considered statistically significant.

## Results

### General characteristics

Overall, we identified 172 *SUFU* PV carriers (83 index patients and 89 relatives) from 83 families, including 127 individuals previously reported.^8 9 14 19 20 23–36 38 39^ In most cases, the *SUFU* PV had been identified in patients with MB either through systematic screening for *SUFU* PV (in 74 index patients, 89%) or because of a familial history of MB (5 families). Another tumour type was the presenting feature in only seven patients: BCC (one patient),^28^ BCC and meningioma (three patients),^30 32 38^ multiple meningiomas (one patient), a bilateral ovarian stromal tumour (one patient) and a pancreatic carcinoma (one patient).^33^ In two patients, the *SUFU* PV was identified in the exploration of a GS phenotype associated with developmental delay but without tumour.

Follow-up data were available for 160 patients. The median age at last follow-up for the whole cohort was 19.5 years (range 0.1–91). At least one tumour has been reported in 117 individuals (68%) while 55 (32%) individuals (including two index patients) were defined as healthy PV carriers, that is, without tumour until their last follow-up (median 38.5 years, range 2–91). Healthy carriers were significantly older at last follow-up than affected patients (median 10 years, range 0.1–85) (p=0.00015).

**Figure 1:**
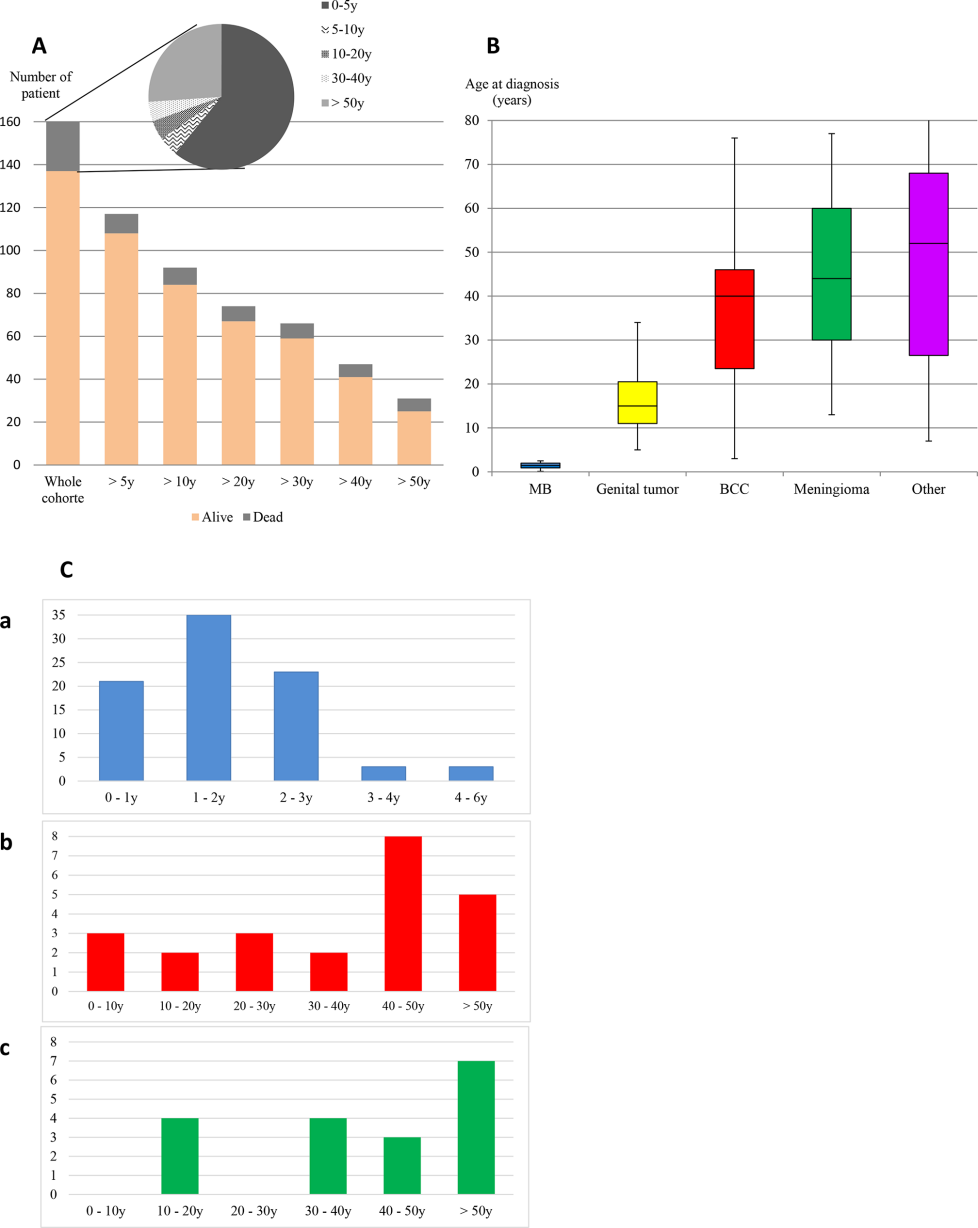
Distribution of age of *SUFU* pathogenic variant carriers. (A) Number of patient exposed to the risk according to the age, (B) patient’s age at diagnosis of tumour onset depending on the type of tumour, (C) patient’s age at diagnosis of tumour onset, for medulloblastoma (MB) (C (a)), for the first basal cell carcinoma (BCC) (C (b)) and for the first meningioma (C (c)).

The age distribution (patient exposed to the risk) and status at the last follow-up are illustrated in figure 1A for 160 patients with available data. At the last follow-up, 137 patients were alive with a median age of 25.5 years (range 0.1–91), and 23 patients died at a median age of 3.5 years (range 0.1–85). Overall, 1–5 malignant or benign tumours were diagnosed in 117 patients, 81/83 (97.5%) index patients and 36/89 (44.5%) relatives. The distribution of tumour types, number of patients and age at diagnosis are described in table 1, figure 1B and online supplemental figure 1. At least one second tumour was diagnosed in 33 patients (28%).

10.1136/jmedgenet-2021-108385.supp1 Supplementary data

**Table 1:** Description of the distribution of the different tumour types in the cohort

Tumour type	Number of patients	Age at diagnosis (years) (median/range)
Medullobastoma	86	1.5 (0.1–5)
BCC*	25	40 (3–76)
Meningioma*	20	44 (13–77)
Gonadal tumours	11	14 (5–34)
Ovarian fibroma (n=4, including one patient with two successive asynchronous fibromas)		
Bilateral ovarian stromal tumour (n=3, including one patient with successive asynchronous tumours)		
Fibrosarcoma (n=1, ovarian; n=1, testicular)		
Bilateral immature teratoma (n=1)^27^		
Bilateral leiomyosarcoma (n=1)^39^		
**Other tumours**		
Sarcoma	2	46, 47, 47, 53
Carcinoma (n=10)		
Breast carcinoma	3	37, 71, 77
Thyroid carcinoma	2	7, 20
Squamous cell carcinoma	2	>50
Pancreatic carcinoma^33^	1	NA
Bladder carcinoma	1	73
Renal cell carcinoma	1	79
Skin hamartoma	3	2, 9, 55
Acute myeloid leukaemia	1	8
Pilocytic astrocytoma	1	20
Myeloma	1	79
Benign ileum myoma	1	42
Thoracic macrocystic lymphangioma	1	At birth

*Age at diagnosis of first BCC/meningioma.

BCC, basal cell carcinoma; NA, not available.

### Medulloblastoma

Overall, 86 patients (74 index patients and 12 relatives) were diagnosed with an MB in 76 distinct families. Multiple cases were diagnosed in six families (online supplemental table 1 and online supplemental figure 2). The MB was discovered during surveillance after presymptomatic screening for only one patient in this cohort.

10.1136/jmedgenet-2021-108385.supp4 Supplementary data

10.1136/jmedgenet-2021-108385.supp2 Supplementary data

MB occurred as a first tumour in all these patients, with a median age at diagnosis of 1.5 years (range 0.1–5.8). MB was diagnosed before the age of 3 in all patients 81/86 (94.2%) but five (5.8%) aged respectively 3.3, 3.4, 3.8, 4 and 5.8 years (figure 1C (a)). Molecular subgrouping and/or histopathological subtype was available for 73/86 patients. All 35 patients with MB with molecular subgrouping belonged to the SHH subgroup. For 39 additional patients, histological subgrouping was concordant with SHH subgroup in 36/39 patients: nodular desmoplastic in 22 or extensive nodularity in 13. In three cases diagnosed before 2010 and with no histological review, the local pathologist reported a classic histology.

The median age at last follow-up was 6 years (range 0.1–37) in 74 patients with MB with follow-up data. Overall, 58/74 (78%) children were alive (median follow-up since diagnosis: 6.0 years, range 0.2–36) with 23 patients older than 10 years at the last follow-up. Sixteen patients died due to progression of the MB in 15 (median time since diagnosis: 1.2 years, range 0–4.3) and because of acute myeloid leukaemia as a second malignancy at age 7.8 years in one. The 5-year OS was 76% (95% CI 64% to 85%) (online supplemental figure 3).

10.1136/jmedgenet-2021-108385.supp3 Supplementary data

A second tumour has been reported in 17 (out 71) patients after the occurrence of the MB, including 13/17 (76%) patients aged 10 years or more at the last follow-up. Among them, nine have received radiotherapy as part of the MB treatment (table 2).

**Table 2:** Patients with secondary tumours after diagnosis of MB

Study	Status and age range at last follow-up	Age range at MB onset (histological type if available)	RT	Secondary malignancies	Other tumour(s)	Number of BCC
Smith *et al* ^8^ Evans *et al* ^19^	Alive (30 s)	Infant (desmoplastic)	Yes	BCC	Meningioma Pilocytic astrocytoma	65
Smith *et al* ^8^ Evans *et al* ^19^	Alive (30 s)	Infant (desmoplastic)	Yes	BCC	Meningioma	3
Smith *et al* ^8^ Evans *et al* ^19^	Alive (20 s)	Infant (desmoplastic)	Yes	BCC	Unilateral ovarian fibroma	11
Taylor *et al* 14 Ng *et al* ^23^	Alive (20 s)	NA (desmoplastic)	Yes	Meningioma		None
Kijima *et al* ^26^	Alive (30 s)	Infant	NA	BCC	Meningioma	NA
Mann *et al* ^28^	Dead (childhood)	Infant (desmoplastic)	NA	BCC infundibulocystic		NA
Mann *et al* ^28^	Dead (infant)	Infant	NA	Skin hamartoma		None
Guerrini-Rousseau *et al* ^31^	Alive (10 s)	Infant (MBEN)	No	Stromal ovarian tumour	Meningioma	None
Guerrini-Rousseau *et al* ^31^	Dead (childhood)	Infant (MBEN)	Yes (PF only)	AML		None
Guerrini-Rousseau *et al* ^31^	Alive (10 s)	Infant (MBEN)	No	Thyroid carcinoma		None
Guerrini-Rousseau *et al* ^31^	Alive (30 s)	Infant (classic)	Yes (CSI)	BCC	Meningioma	>20
Guerrini-Rousseau *et al* ^31^	Alive (10 s)	Infant (desmoplastic)	No	Hamartoma		None
Ogden *et al* ^39^	Alive (30 s)	Infant	Yes (CSI)	Bilateral ovarian leiomyosarcoma	BCC Meningioma	>100
Present report	Alive (20 s)	Infant	No	Bilateral ovarian stromal tumour		None
Present report	Alive (childhood)	Infant (desmoplastic)	No	Ovarian fibroma	Controlateral ovarian fibroma	None
Present report	Alive (10 s)	Infant	Yes (CSI)	Meningioma	Meningioma	None
Present report	Alive (20 s)	Infant (MBEN)	Yes (CSI)	Meningioma	Ovarian fibrosarcoma Thyroid carcinoma	None

Age range at last follow-up (2.2–37 years), age range at MB onset (0.5–2.5 years), age range at diagnosis of the other tumours (7–35 years)

AML, acute myeloid leukaemia; BCC, basal cell carcinoma; CSI, craniospinal radiotherapy; MB, medulloblastoma; MBEN, medulloblastoma with extensive nodularity; NA, not available; PF, posterior fossa; RT, radiotherapy.

### Basal cell carcinomas

BCC was reported in 25 patients (11 index patients and 14 relatives). The median age at diagnosis of the first BCC was 40 years (range 3–76) (figure 1C (b)). Only five patients were diagnosed with a BCC before the age of 20 years, all of them as a second malignancy. The number of BCCs is available for 18 patients (median age at last follow-up was 40.5 years). Half of them (aged 9–52 years at diagnosis of first BCC and 31–79 years at last follow-up) developed >20 BCC.

BCCs occurred as the first tumour in 16 patients, all occurring after the age of 20 years (median age of 43 years, range 22–76). Most patients (11/16) were identified through a systematic screening for *SUFU* PV in GS cohorts and have already been reported.^8 19 28 30 32^ All patients but eight developed at least one other tumour before (11 tumours in 9 patients, including 7 MBs, 2 sarcomas, 1 ovarian tumour and 1 meningioma) or after BCC diagnosis (18 tumours in 15 patients). Among the 23 MB survivors aged 10 years or more at the last follow-up, 6 patients (26%) developed a BCC with a median time between the diagnosis of MB and BCC of 16.8 years (range 7–26). Of those with available data, 5/5 patients were treated with radiotherapy (table 2).

### Meningiomas

Twenty patients from 13 different families have been reported with a meningioma (13 index patients and 7 relatives); all were intracranial. They were described as a first, second, third and fourth tumour for four, nine, six and one patient(s). Meningiomas have been reported in members of a large family with several cases,^27^ in patients treated for MB or in their relatives, or in patients with clinical features suggestive of GS. Seven patients developed multiple meningiomas, including the five familial cases reported in 2012.^27^ The median age at diagnosis of the first meningioma was 44 years (range 13–77) (figure 1C (c)). Eight patients had a meningioma before the age of 35 years (median age of 24.5 years, range 13–35), occurring after radiotherapy for an MB in the 6/7 patients with data on MB treatment (table 2). Only one patient developed a meningioma 12 years after the occurrence of an MB treated without radiotherapy.

### Gonadal and other tumours

Eleven patients (seven index patients and four relatives) were diagnosed with a gonadal tumour, which occurred as a first tumour in four cases. Ten tumours were classified as a sexual cord or stromal tumours: five ovarian fibromas, four ovarian stromal tumours and one tumour diagnosed as an ovarian fibrosarcoma (sexual cord tumour with clear evidence of a fibrothecoma with malignant features). One bilateral immature teratoma^27^ and one bilateral leiomyosarcoma^39^ were previously reported. One relative was reported with a testicular fibrosarcoma at the age of 10 years. Overall, bilateral ovarian tumours were observed in six patients. These gonadal tumours mostly occurred at paediatric age, with a median age at diagnosis of 14 years (range 5–34). The four ovarian stromal tumours, which occurred in three girls, were bilateral in all the cases, synchronous for two of them and subsequent for the last patient. In cases where treatment data were available, surgery was performed in all, associated with chemotherapy in two.

Several other tumours were reported, as shown in table 1. No cardiac fibromas were observed.

### Risk of tumours in relatives

**Figure 2:**
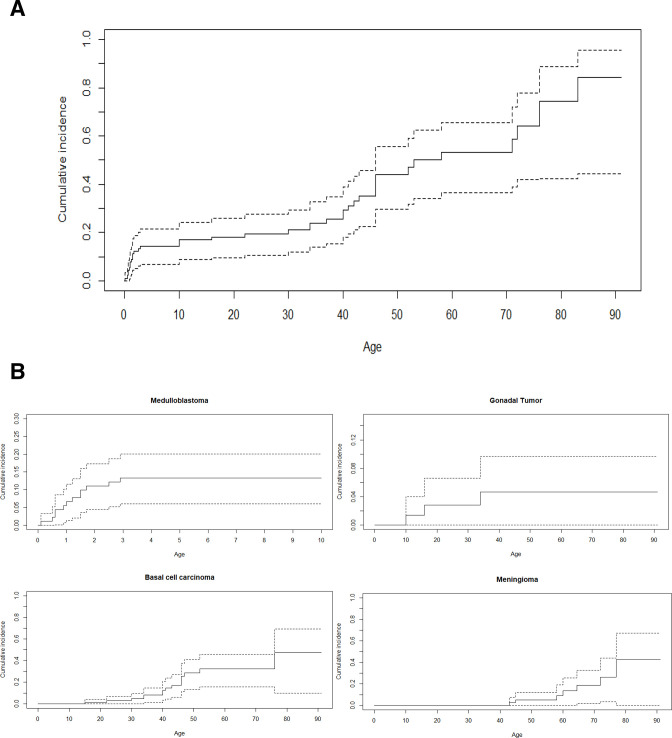
Estimated cumulative incidence curves of tumours, among relatives carrying a *SUFU* pathogenic variant, according to the Nelson-Aalen estimator, with 95% CIs represented as dotted lines. (A) For all tumours. (B) For each main tumour type: medulloblastoma, basal cellcarcinoma, meningioma and genital tumour.

*SUFU* PV inheritance could be tested in 41 of 83 families. The PV was de novo in 11 patients (27%) and inherited in 30/41 families (73%). In these 30 families, 119 mutation carriers have been identified: 30 index and 89 relatives. We could analyse the occurrence of tumours in 89 relatives in whom the median age at last follow-up was 40.5 years (range 0.1–91). A total of 53 individuals (60%) were alive without a tumour at a median age of 37 years (range 1–91), and 36 patients (40%) developed a total of 55 tumours, some of them after the age of 50 years. The median age at the occurrence of the first tumour was 34 years. The cumulative incidence of any tumour at the age of 5, 20 and 50 years was, respectively, 14.4% (95% CI 6.8 to 21.4), 18.2% (95% CI 9.7 to 25.9) and 44.1% (95% CI 29.7 to 55.5) (figure 2A). The cumulative incidence of MB was 13.3% (95% CI 6 to 20.1) at 5 years and remained stable afterwards. The cumulative incidence of gonadal tumours was 2.8% (95% CI 0 to 6.6) and 4.6% (95% CI 0 to 9.7) at 20 and 50 years, respectively. The cumulative incidence of BCC and meningioma at 50 years was 28.5% (95% CI 13.4 to 40.9) and 5.2% (95% CI 0 to 12) (figure 2B).

### Germline *SUFU* PV analysis

**Figure 3:**
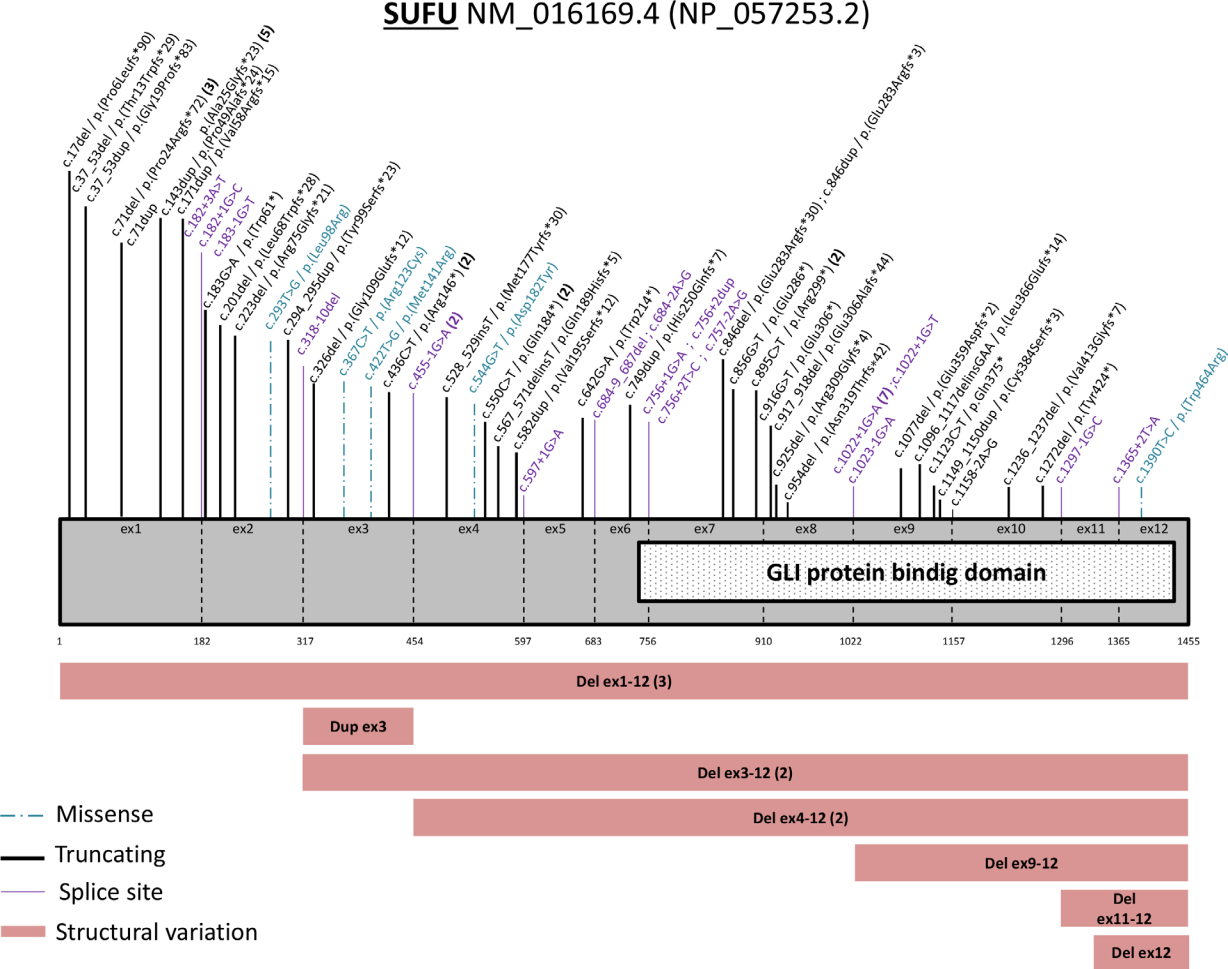
Representation of all the pathogenic or likely pathogenic variants described on the *SUFU* gene.

Overall, 64 different germline *SUFU* PVs were identified in 83 families, reported across the entire *SUFU* gene (figure 3). Nine different variants were identified in 28 families (online supplemental table 3). We identified 24 frameshift variants (37%), 9 nonsense variants (14%), 5 missense variants (8%), 19 splice site variants (30%) and 7 different structural variations involving either the complete *SUFU* gene, several exons or just one exon (11%). Nucleotide position 71 in the first exon (3 deletions of the cytosine and 5 duplications at that position) was found to be mutated in 8 families and the c.1022+1G>A variant which has been shown to result in the skipping of exon 8^14^ was the splice PV the most frequently reported (in 7 families). Three patients with a severe intellectual disability have large structural variants that could be associated with a contiguous gene syndrome. There was not a significantly higher risk of MB according to the type of variation, the expected protein effects (structural variations, nonsense and frameshift PVs vs missense and splice PVs) (p=0.8728, χ^2^ test) or the involvement of the PV in the DNA binding domain of the *SUFU* gene (p=0.0827, χ^2^ test) (online supplemental table 4).

10.1136/jmedgenet-2021-108385.supp5 Supplementary data

10.1136/jmedgenet-2021-108385.supp6 Supplementary data

## Discussion

Analysing together data from 48 unpublished patients and all cases previously reported allowed us to constitute a large series of germline *SUFU* PV carriers in which we could analyse tumour occurrence. In such a rare situation where presymptomatic testing is not proposed as a routine procedure, a precise evaluation of tumour risk cannot be performed. As most index patients in this series have been identified after the occurrence of a malignancy, the evaluation of tumour risk on the entire population overestimates the risk. In order to reduce this ascertainment bias, we analysed the risk of tumour in relatives only after excluding the index cases. Nevertheless, a residual ascertainment bias related to the selection of relatives from families characterised by the presence of an affected case cannot be excluded. However, data from this series allow describing the oncological spectrum and risk associated with germline *SUFU* PV and the period of onset of each tumour type during life, which is the most important information for designing guidelines for PV carriers follow-up.

We confirmed that the tumour penetrance is high, although incomplete reaching 44% at 50 years. It cannot be compared with *PTCH1* PV carriers since such an evaluation of cancer incidence in relatives is not available in this population. The overall tumour risk in the entire population, including index patients, reaches 68%. This is in the range of the risk described in *PTCH1*-associated GS, where tumour risk has been estimated around 55%–60%.^19^ In addition, we could confirm that the spectrum of *SUFU*-associated GS differs from *PTCH1-*associated GS as previously suggested in smaller studies.^19 31^

The most frequent tumour is MB. It affects 89% of index patients. This rate is clearly higher than in patients carrying a germline *PTCH1* PV in whom MB incidence has been reported to be 2%.^19^ The cumulative incidence of MB in relatives reaches 13.3%. This risk may be slightly overestimated since several families were tested after the occurrence of MB in siblings but is lower than previously estimated.^19 25^ As previously reported, all MBs were classified in the SHH subgroup^20 34^ and most of them with desmoplastic/nodular histology.^25^ We confirmed that the occurrence of MB was mainly limited to the first 3 years of life, with only 5/86 patients (5.8%) occurring after age 3 years but before 6 years.

Gonadal tumours mostly occur in children and teenagers with a median age at diagnosis of 14 years (range 5–34). In the Manchester cohort, systematic ultrasound in individuals with GS led to the detection of an ovarian tumour in 3/7 females (43%) with germline *SUFU* PV, compared with 4/68 females (5.9%) with *PTCH1* PV.^19^ In the present series, the cumulative incidence of ovarian tumours has been estimated at around 10% at 50 years in females (4.6% in the entire population). The occurrence of malignant stromal tumours, which have not been reported yet in *PTCH1*-related GS where only fibromas have been reported, has to be underlined.

The cumulative incidence of meningioma at 50 years is about 5%. Nevertheless, meningioma seems to be more frequent in *SUFU* PV carriers (11%) than in the Manchester cohort of 126 patients with GS associated with *PTCH1* germline PVs in whom the incidence of meningiomas was <2%.^19^ In contrast, the risk of BCC for *SUFU* PV carriers is clearly lower than for those with a *PTCH1* PV with only 11/31 (35%) *SUFU* patients >50 years affected with BCC. The cumulative incidence is 28.5% at 50 years of age in this series compared with 76.5%–80% at 50 years in *PTCH1* PV carriers.^3^ The occurrence of BCC or meningioma in germline *SUFU* PV carriers seems to be similar with a double peak occurrence of onset. As a first oncological event, apart from any previous treatment by radiotherapy or chemotherapy, the onset of these tumours seems to occur in a few patients (about 5%), mainly in adults around 40 years. In children treated for MB and exposed to chemotherapy and/or radiotherapy, the risk of BCC and meningioma is higher, and the onset is earlier (before the age of 30 years). It is noteworthy that neither odontogenic keratocysts nor cardiac fibromas occurred in the present series, with both tumours being hallmarks of *PTCH1*-associated GS.^19^

The risk of multiple tumours in *SUFU* PV carriers is clearly high, affecting 28% (33/117) of patients who developed a first tumour. The risk of second neoplasms, especially BCC and meningioma, after treatment of an MB can only be assessed for the first years following treatment since the follow-up is still short in most patients. However, since 12/23 (52%) patients aged >10 years at last follow-up after MB diagnosis have developed at least one secondary malignancy, this risk is clearly much higher than in an unselected series of MB in which a rate of secondary primary tumours of 3.1% at 10 years has been reported.^40^ This high incidence warrants specific guidelines for the follow-up of these patients.^41^ It is noteworthy that one-third of meningioma reported in this study occurred in patients previously treated with cranial radiotherapy for an MB. The 5-year OS of patients treated for an MB was 76% and is in the range of survival rates described in a large series of young children either with nodular desmoplastic MB (5-year OS=89% and 81% for M0 and M+ patients, respectively)^42^ or SHH-MB in infants (5-year OS=62%).^43^ With the relatively short follow-up of the patients with MB in this cohort, most of the secondary tumours observed were not life-threatening. Except for one case, death was always linked to MB progression. Given the incidence of germline mutations in young patients with MB^20^ and the consequences of the presence of a germline variant on care and follow-up, genetic testing for *SUFU* and *PTCH1* is of paramount importance in all children with SHH-MB before the age of 5 years. The best therapeutic strategy aiming to keep this high survival rate while sparing patients of the risk of second malignancy has to be evaluated. Upfront radiation sparing approaches could be justified given the expected high cure rate of SHH-MB in infants even in the absence of radiation.^42^ The high risk of a secondary tumour in *SUFU* PV carriers treated for MB warrants early detection of BCC and meningioma, even in the absence of irradiation. Presymptomatic testing should be offered to the relatives during family genetic counselling to allow appropriate tumour surveillance.^41^

Because of this new insight into the *SUFU*-related cancer spectrum and risks requiring specific management, we think the clinical condition associated with *SUFU* PVs should be described as a specific syndrome requiring specific management. In contrast, the term ‘GS’ should be restricted to clinical manifestation associated with *PTCH1* PVs, acknowledging that these syndromes overlap. Testing for a germline *SUFU* mutations should be proposed in all patients presenting with a tumour belonging to *SUFU* spectrum (SHH-MB, BCC, meningioma, ovarian stromal or fibrous tumour) and for whom genetic predisposition is suspected because of young age at diagnosis, a familial history of cancer of multiple tumours. Recommendations for cancer surveillance in GS already published,^4 44^ were recently adapted by the European Host Genome Working Group to the genetic background (*PTCH1* or *SUFU* PV) with the support of data presented here^41^ (table 3). As the risk of MB is 13.3% at the age of 5 years, early postnatal testing for a *SUFU* PV can be offered because the result of the analysis will have a major impact on the surveillance of children with *SUFU* PV in the first years of life.

**Table 3:** Screening recommendations for patients with *SUFU* germline pathogenic variations

Tumours	Methods for screening	Surveillance recommendations in carriers of germline *SUFU* variants (periodicity, age to start and to end)
BCC	Dermatological examination	Annually beginning at age 20 years
		earlier if previous radiotherapy
Medulloblastoma	Brain MRI without contrast agent except for the first MRI	Every 3–4 months during the first 3 years then every 6 months until 5 years
Meningioma	Brain MRI	Every 3–5 years beginning at age 30 years for patients with no previous MB
		after healing of the MB in other patients
Ovarian tumour	Pelvic ultrasound	Every 3 years, beginning at age 5 years
Cardiac fibroma	Echocardiogram	At the time of diagnosis of GS, ideally in the first 6 months of life

BCC, basal cell carcinoma; GS, Gorlin syndrome; MB, medulloblastoma.

This large study also described molecular data of germline *SUFU* PVs and highlighted a certain level of recurrence for some variants. Apart from structural variants that could be associated with a contiguous gene syndrome, no significant genotype-phenotype associations could be identified.

In conclusion, germline *SUFU* PV carriers have a life-long increased risk of tumours with a spectrum dominated by medulloblastoma before the age of 5 years, gonadal tumours during adolescence and BCC and meningioma in adulthood, justifying fine-tuned surveillance programmes, and the identification of healthy mutation carriers among relatives.

## Data Availability

All data relevant to the study are included in the article or uploaded as supplementary information.
