# Supplementary figures and images for: Cancer risk and tumour spectrum in 172 patients with a germline SUFU pathogenic variation: a collaborative study of the SIOPE Host Genome Working Group
Source: J Med Genet. 2022 Jun 29;59(11):1123–32. doi: 10.1136/jmedgenet-2021-108385 (PMC9613872; doi:10.1136/jmedgenet-2021-108385)

Figure S1

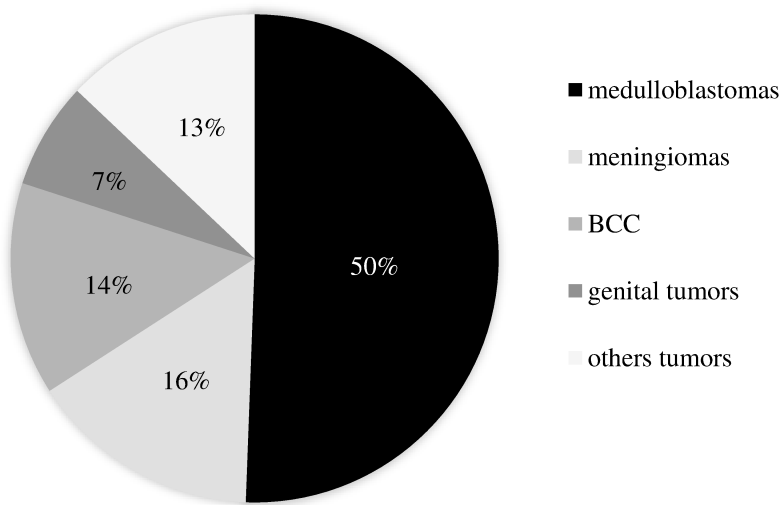

**Figure S1.** Distribution of tumor types in the cohort

Supplement: Supplementary data [file jmedgenet-2021-108385supp001.pdf]

Figure S3

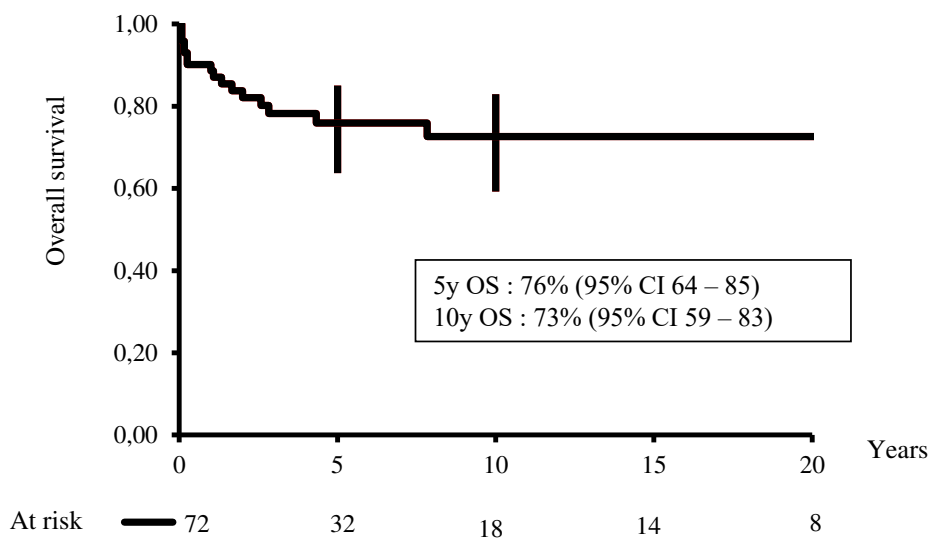

**Figure S3.** Overall survival after a medulloblastoma of children with germline *SUFU* PV (n=72).

Supplement: Supplementary data [file jmedgenet-2021-108385supp003.pdf]
