# Supplementary material for: Cancer risk and tumour spectrum in 172 patients with a germline SUFU pathogenic variation: a collaborative study of the SIOPE Host Genome Working Group
Source: J Med Genet. 2022 Jun 29;59(11):1123–32. doi: 10.1136/jmedgenet-2021-108385 (PMC9613872; doi:10.1136/jmedgenet-2021-108385)
Supplement: Supplementary data [file jmedgenet-2021-108385supp004.pdf]

BMJ Publishing Group Limited (BMJ) disclaims all liability and responsibility arising from any reliance placed on this supplemental material which has been supplied by the author(s)

Table S1

Family	c. mutation type	Index patient or relative	MB subgroup	Age at diagnosis (years)
Family 1	c.37_53dup	Index patient	MB,SHH	0.4
		Relative (sibling)	MB (NA)	NA
Family 2*	c.756+1G>A	Relative (sibling)	ND MB	0.6
		Relative (sibling)	MB (NA)	0.5
Family 3	c.71dup	Index patient	MBEN	0.9
		Relative (cousin)	ND MB	2.9
		Relative (cousin)	ND MB	1.7
		Relative (aunt)	MB (NA)	0.6
		Relative (cousin)	MB (NA)	1.2
Family 4	c.71del	Index patient	MBEN	0.3
		Relative (sibling)	MBEN	0.1
Family 5	c.567_571delinsT	Index patient	MBEN	2.2
		Relative (sibling)	Classic MB	2.5
		Relative (sibling)	Classic MB	1.5
Family 6	c.201delC	Index patient	MB (NA)	2.8
		Relative (cousin)	MB (NA)	0.9

Table S1. Family history with multiple children with a germline *SUFU* PV affected with medulloblastoma in the same family
* Index patient affected with BCC (28)
