# Supplementary material for: Cancer risk and tumour spectrum in 172 patients with a germline SUFU pathogenic variation: a collaborative study of the SIOPE Host Genome Working Group
Source: J Med Genet. 2022 Jun 29;59(11):1123–32. doi: 10.1136/jmedgenet-2021-108385 (PMC9613872; doi:10.1136/jmedgenet-2021-108385)
Supplement: Supplementary data [file jmedgenet-2021-108385supp002.pdf]

Figure S2

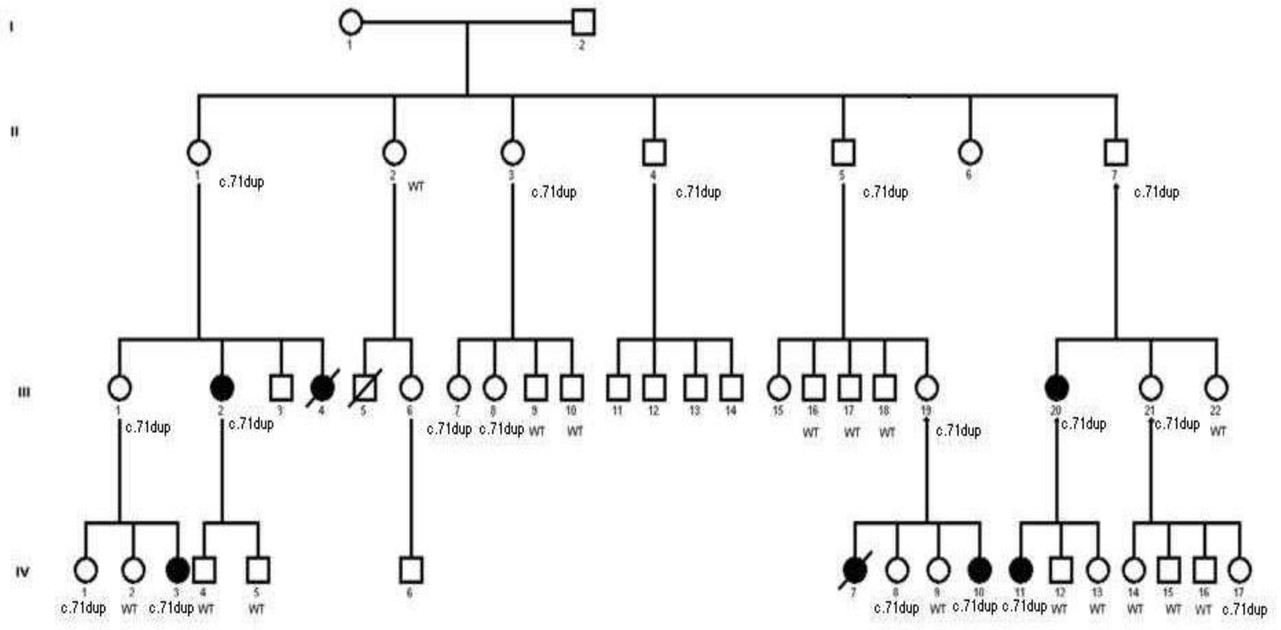

Figure S2. Genealogical table of one family with an history of 5 children (III-2, III-20, IV-3, IV-10 and IV-11) with a documented germline *SUFU* PV affected with a proven medulloblastoma (Family 3)

Legend: Girls affected with medulloblastoma (probable or proven) are shown with filled circles.
