# Supplementary material for: Cancer risk and tumour spectrum in 172 patients with a germline SUFU pathogenic variation: a collaborative study of the SIOPE Host Genome Working Group
Source: J Med Genet. 2022 Jun 29;59(11):1123–32. doi: 10.1136/jmedgenet-2021-108385 (PMC9613872; doi:10.1136/jmedgenet-2021-108385)
Supplement: Supplementary data [file jmedgenet-2021-108385supp005.pdf]

Table S2

Table S2. Description of the various *SUFU* PVs identified in the cohort (NM_016169.4)

publication	mutation c	mutation p	Mutation type	Recurrence in n families
(9,24,31, 34) (5 families) and present report (2 families)	c.1022+1G>A	p.?	Splicing	7 families
(31,35) (2 families) and present report (3 families)	c.71dup	p.(Ala25Glyfs*23)	Frameshift	5 families
(20,31) (3 families)	c.71del	p.(Pro24Argfs*72)	Frameshift	3 families
(14,29) and present report	c.1-?_1455+?del	del exon 1-12	Structural variation	3 families
(8,19, 31)	c.318-?_1455+?del	del exon 3-12	Structural variation	2 families
(8,19,26)	c.550C>T	p.(Gln184*)	Nonsense	2 families
(20) and present report	c.436C>T	p.(Arg146*)	Nonsense	2 families
(20) and present report	c.455-1G>A	p.?	Splicing	2 families
(20) and present report	c.895C>T	p.(Arg299*)	Nonsense	2 families
(8,19)	c.544G>T	p.(Asp182Tyr)	Missense	1 family
(20)	c.1077del	p.(Glu359Aspfs*2)	Frameshift	1 family
(20)	c.37_53del	p.(Thr13Trpfs*29)	Frameshift	1 family
(20)	c.37_53dup	p.(Gly19Profs*83)	Frameshift	1 family
(20)	c.642G>A	p.(Trp214*)	Nonsense	1 family
(20)	c.684-2A>G	p.?	Splicing	1 family
(20)	c.749dup	p.(His250Glnfs*7)	Frameshift	1 family
(20)	c.925del	p.(Arg309Glyfs*4)	Frameshift	1 family
(14,23)	c.143dup	p.(Pro49Alafs*24)	Frameshift	1 family
(14,23)	c.183-1G>T	p.?	Splicing	1 family
(24)	c.846dup	p.(Glu283Argfs*3)	Frameshift	1 family
(27)	c.367C>T	p.(Arg123Cys)	Missense	1 family
(28)	c.756+1G>A	p.?	Splicing	1 family
(30)	c.757-2A>G	p.?	Splicing	1 family
(31)	c.1023-?_1455+?del	del exon 9-12	Structural variation	1 family
(31)	c.1096_1117delinsGAA	p.(Leu366Glufs*14)	Frameshift	1 family
(31)	c.1123C>T	p.(Gln375*)	Nonsense	1 family
(31)	c.1149_1150dup	p.(Cys384Serfs*3)	Frameshift	1 family
(31)	c.1297-1G>C	p.?	Splicing	1 family
(31)	c.182+3A>T	p.?	Splicing	1 family
(31)	c.294_295dup	p.(Tyr99Serfs*23)	Frameshift	1 family
(31)	c.318-?_454+?dup	dup exon 3	Structural variation	1 family
(31)	c.318-10del	p.?	Splicing	1 family
(31)	c.422T>G	p.(Met141Arg)	Missense	1 family
(31)	c.567_571delinsT	p.(Gln189Hisfs*5)	Frameshift	1 family
(32)	c.954del	p.(Asn319Thrfs*42)	Frameshift	1 family
(33)	c.223del	p.(Arg75Glyfs*21)	Frameshift	1 family
(34)	c.1023-1G>A	p.?	Splicing	1 family
(34)	c.171dup	p.(Val58Argfs*15)	Frameshift	1 family
(34)	c.528_529insT	p.(Met177Tyrfs*30)	Frameshift	1 family
(34)	c.684-9_687del	p.?	Splicing	1 family
(34)	c.916G>T	p.(Glu306*)	Nonsense	1 family
(36)	c.1272del	p.(Tyr424*)	Nonsense	1 family
(38)	c.1365+2T>A	p.Ile433_Glu455del	Splicing	1 family
(39)	c.597+1dupG	p.?	Splicing	1 family
Present report	c.1022+1G>T	p.?	Splicing	1 family
Present report	c.1158-2A>G	p.?	Splicing	1 family
Present report	c.1236_1237del	p.(Val413Glyfs*7)	Frameshift	1 family
Present report	c.1297-?_1455+?del	del exon 11-12	Structural variation	1 family
Present report	c.1366-?_1455+?del	del exon 12	Structural variation	1 family
Present report	c.1390T>C	p.(Trp464Arg)	Missense	1 family
Present report	c.17del	p.(Pro6fs*89)	Frameshift	1 family
Present report	c.182+1G>C	p.?	Splicing	1 family
Present report	c.183G>A	p.(Trp61*)	Nonsense	1 family
Present report	c.201del	p.(Leu68Trpfs*28)	Frameshift	1 family
Present report	c.293T>G	p.(Leu98Arg)	Missense	1 family
Present report	c.326del	p.(Gly109Glufs*12)	Frameshift	1 family
Present report	c.455-?_1455+?del	del exon 4-12	Structural variation	1 family
Present report	c.582dup	p.(Val195Serfs*12)	Frameshift	1 family
Present report	c.597+1G>A	p.?	Splicing	1 family
Present report	c.756+2dup	p.?	Splicing	1 family
Present report	c.756+2T>C	p.?	Splicing	1 family
Present report	c.846del	p.(Glu283Argfs*30)	Frameshift	1 family
Present report	c.856G>T	p.(Glu286*)	Nonsense	1 family
Present report	c.917_918del	p.(Glu306Alafs*44)	Frameshift	1 family
