# Supplementary material for: Cancer risk and tumour spectrum in 172 patients with a germline SUFU pathogenic variation: a collaborative study of the SIOPE Host Genome Working Group
Source: J Med Genet. 2022 Jun 29;59(11):1123–32. doi: 10.1136/jmedgenet-2021-108385 (PMC9613872; doi:10.1136/jmedgenet-2021-108385)
Supplement: Supplementary data [file jmedgenet-2021-108385supp006.pdf]

BMJ Publishing Group Limited (BMJ) disclaims all liability and responsibility arising from any reliance placed on this supplemental material which has been supplied by the author(s)

# Table S3

| | **p** |
|---|---|
| Frameshift (n=84) versus other (n=88) | 0.169796 |
| Missense (n=14) versus other (n=158) | 0.163241 |
| Nonsense (n=14) versus other (n=158) | 0.050943 |
| Splice (n=46) versus other (n=126) | 0.605297 |
| Structural variation (n=14) versus other (n=158) | 0.402844 |
| Structural variation, nonsense or frameshift PVs (n=112) versus missense or splicing PVs (n=60) | 0.872893 |

**Table S3.** Risk of medulloblastoma according to the *SUFU* PV. Chi-quare test depending on the type of variation or the expected protein effects of the *SUFU* gene

p-values <0.05 were considered statistically significant

PVs : pathogenic variants
